# A Tool for Shared Decision Making on Referral for Prostate Biopsy in the Primary Care Setting: Integrating Risks of Cancer with Life Expectancy

**DOI:** 10.3390/jpm9020019

**Published:** 2019-04-22

**Authors:** Jan F.M. Verbeek, Daan Nieboer, Chris Parker, Michael W. Kattan, Ewout W. Steyerberg, Monique J. Roobol

**Affiliations:** 1 Department of Urology, Erasmus University Medical Center, 3000 CA Rotterdam, The Netherlands; d.nieboer@erasmusmc.nl (D.N.); m.roobol@erasmusmc.nl (M.J.R.); 2 Department of Public Health, Erasmus Medical Center, 3000 CA Rotterdam, The Netherlands; E.W.Steyerberg@lumc.nl; 3 Department of Academic Urology, Royal Marsden Hospital, Sutton SM2 5PT, UK; chris.parker@icr.ac.uk; 4 Department of Quantitative Health Sciences, Cleveland Clinic, Cleveland, OH 44195, USA; kattanm@ccf.org; 5 Department of Biomedical Data Sciences, Leiden University Medical Center, 2333 ZC Leiden, The Netherlands

**Keywords:** prostate cancer, screening, prostate cancer survival, prediction model, mortality, treatment, life expectancy

## Abstract

Prostate cancer (PCa) testing involves a complex individually based decision making process. It should consider competing risks from other comorbidities when estimating a survival benefit from the early detection of clinically significant (cs)PCa. We aimed to develop a prediction tool that provides concrete advice for the general practitioner (GP) on whether to refer a man for further assessment. We hereto combined the probability of detecting csPCa and the potential overall survival benefit from early detection and treatment. The PCa detection probabilities were derived from 3616 men enrolled in the Dutch arm of the European Randomized Study of Screening for Prostate Cancer (ERSPC). Survival estimates were derived from 19,834 men from the Surveillance, Epidemiology, and End Results (SEER) registry, ERSPC, and Dutch life tables. Treatment benefit was estimated from the Prostate Cancer Intervention versus Observation Trial (PIVOT, *n* = 731). The prediction of csPCa detection was based on prostate-specific antigen (PSA), age, %freePSA, and digital rectal examination (DRE). The life expectancy (LE) for patients with PCa receiving no treatment was adjusted for age and Charlson comorbidity index. A negative impact on LE and treatment benefit was found with higher age and more comorbidity. The proposed integrated approach may support triage at GP practices, as PCa is a heterogeneous disease in predominantly elderly men.

## 1. Introduction

Prostate-specific antigen- (PSA) based screening for prostate cancer (PCa) can reduce PCa mortality, as has been demonstrated in a large-scale European randomized screening trial [1]. However, PSA-based screening also results in the detection of considerable numbers of indolent PCa due to lack of risk stratification and the random method of sampling. This results in over-diagnosis and overtreatment of clinically harmless PCa negatively affecting the harm–benefit ratio [2]. Therefore, referral for further testing should only be applied to patients with high risk of metastasis and cancer-related mortality. However, this ideal risk stratification is not yet feasible, even with the use of novel techniques such as imaging and contemporary biomarkers. The U.S. Preventative Services Task Force, European Association of Urology (EAU), and American Urological Association (AUA) guidelines recommend that men aged 55–69 years should be informed about the benefits and the harms of screening, and PSA testing should be offered only after informed choice [3,4,5]. For most men, PCa screening starts with a visit to the general practitioner (GP). It is the GP’s task/challenge to guide men and to identify men who can benefit from early detection and treatment. To assist (future) patients and physicians in interpreting the clinical significance of PSA levels, multivariable PCa risk calculators (RC) have been developed that estimate the probability of detecting (potentially aggressive) PCa if referred for prostate biopsy. These RCs improve predictions by including other relevant information, such as age or family history, in addition to PSA levels [6,7,8]. However, these PCa RCs do not include a patient’s characteristics, e.g., life expectancy (LE) and long-term effects of treatment. These are relevant factors, since risk of experiencing harm from a potentially aggressive PCa is likely to be offset, to some extent, by a reduced LE for older men [9]. To obtain insight in these competing risks, they need to be quantified and modeled. The aim of this study is to provide a tool suitable for use in primary care that, on the basis of readily available information, can assess the risk of having a potentially aggressive PCa in the context of a man’s LE, and in addition, quantifies potential treatment benefit.

## 2. Materials and Methods

**Figure 1:**
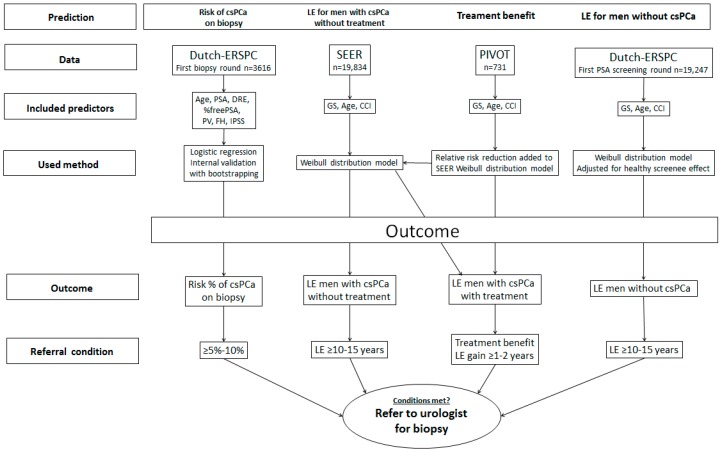
Flowchart of development of prediction model predicting risk of csPCa on current biopsy, overall life expectancy, and treatment benefit for each individual patient. LE: life expectancy, csPCa: clinically significant prostate cancer.

Several aspects should be taken into account in a shared decision making process to refer for a biopsy: first, an individual’s current risk of having clinically significant PCa (csPCa) [International Society of Urological Pathology (ISUP) grade ≥2]; second, his LE in the absence of csPCa; third, his LE in the case of undetected and untreated csPCa; and lastly, how much benefit could be gained from treatment in the case of csPCa diagnosis? Since there is no single dataset available comprehensive enough to simultaneously assess these individual probabilities, multiple data sources were used for the development of the proposed tool. Figure 1 provides an overview of the different prediction models and their underlying sources. To summarize, the model predicting the presence of csPCa at the time of biopsy was based on the Dutch arm of the European Randomized Study of Screening for Prostate Cancer (ERSPC). Estimates on LE of men diagnosed with csPCa receiving no active treatment were based on the Surveillance, Epidemiology, and End Results (SEER) registry. The benefit of active treatment was estimated from the Prostate Cancer Intervention versus Observation Trial (PIVOT). To predict the LE for men with csPCa receiving active treatment, the treatment benefit from the PIVOT was added to the LE prediction from the SEER. Estimates on LE for men without csPCa were predicted using data from the Dutch ERSPC. The outcomes of the different prediction models are displayed in an easy-to-read format that enables evaluation of csPCa risk in the context of a man’s LE and treatment effect. Final recommendations for GPs formulated as “no referral needed” or “refer to a urologist” were based on consensus of risk thresholds by the Prostate Cancer United Kingdom Prostate Risk Working Group (PCUK-RWG) [10], taking into account the probabilities for having csPCa on a current biopsy (>5–10%), life expectancy (>10–15 years), and treatment benefit (1–2 years additional gain in overall survival). If the calculated risk is below the lower limit, the advice is not to refer. If the risk is within range (see Figure 1), the patient’s preferences can be dictated. If calculated risks are above the upper limit, the patient should definitely be referred. It should be noted that referral to secondary care is also indicated when multiple criteria are above the given range. Here, the risk of csPCa and the potential treatment benefit should be leading, even with an LE estimated to be below 10 years. For example, if a patient would have an elevated risk of having csPCa when biopsied, an estimated LE of nine years without csPCA, but a potential treatment benefit of three years, he should be referred for biopsy, and when the suspicion of csPCa is confirmed, he should be actively treated. The analysis for each prediction model is described in detail below. 

The risk of having a biopsy-detectable csPCa is based on 3616 men who received transrectal ultrasound-guided sextant biopsies in the first screening round of the Dutch arm of the ERSPC [7]. Only variables to which a GP has easy access were included in the analyses, i.e., age at time of biopsy, PSA (two log centered), %freePSA (freePSA/total PSA; two log centered), results of the digital rectal examination (DRE) including a rough estimate of prostate volume (PV) estimated during DRE (25, 40, or 60 cc; two log centered [11]), family history, and the International Prostate Symptom Score (IPSS). These predictors were combined in a series of logistic regression models in which the discriminative ability of each model was assessed. First, the model was fitted to all observations in the given set, and the concordance index was calculated. Second, a dataset was formed by bootstrapping with 1000 samples in which the model was again developed and then validated based on the original data. The difference in performance between the original and the bootstrapped data was the estimated “optimism”. The models with the highest concordance index after correction for optimism were selected. As the concordance index does not reflect calibration, the Index of Prediction Accuracy (IPA) considering both discrimination and calibration was calculated; a higher IPA indicated more accurate predictions [12]. The clinical utility of the models was expressed with net benefit (NB) by summing the benefits (true positive biopsies) and subtracting the harms (unnecessary biopsy). The harms were weighted by a factor related to the relative harm of a missed cancer versus unnecessary biopsies [13]. This weighting was derived from the threshold probability for csPCa at which a patient would opt for a biopsy (range considered 3–10%) and were displayed in a decision curve analysis graph. A model was considered to be clinically useful if its NB was higher than the default strategy (biopsy if PSA ≥ 3.0 ng/mL). 

The LE for men with csPCa without receiving treatment was estimated based on the SEER program [14]. The SEER consisted of 19,639 men (age ≥65) diagnosed in the period from 1 January 1992 to 31 December 2005 and 195 men (age <65) diagnosed between 1 January 1971 and 31 December 1984 [15,16]. The SEER reported overall mortality outcomes for Gleason Score (Gleason Score 5–7 and Gleason 8–10), age (55–59, 60–64, 65–69, 70–74, 75+) and Charlson comorbidity index (0, 1, ≥2). These survival curves were approximated using a Weibull distribution. To estimate LE for men with Gleason 3 + 4 or higher (ISUP grade ≥2), the Gleason Score distribution from the Dutch ERSPC was used to adjust the SEER’s reported Gleason Score distribution [17]. 

Also, a relative effect of 0.79 on PCa mortality was applied to the pre-PSA era SEER cohort to include the reduction in PCa mortality by the introduction of PSA [16,18]. The reported SEER’s outcomes and adjustments were fitted in a model with a Weibull distribution to predict individual LE for men with csPCa receiving no treatment. 

The treatment benefit of csPCa was based on the PIVOT [19]. The PIVOT is a randomized trial comparing treatment effect of radical prostatectomy versus watchful waiting in 731 men with localized prostate cancer diagnosed in the era of PSA testing. The relative effect for all-cause mortality for treatment versus no active treatment was extracted, and the life expectancy for men with csPCa and treatment were estimated using the survival curves, which were adjusted using the relative treatment effect from the PIVOT trial. Besides the PIVOT study, other randomized clinical trials comparing PCa treatment with observation include the Scandinavian Prostate Cancer Group Study Number 4 (SPCG-4) and the Prostate Testing for Cancer and Treatment (ProtecT) trial [20,21]. These trials have similar long follow-up. A sensitivity analysis was performed to compare the LEs after treatment for men with csPCa using the relative treatment effects from the PIVOT, SPCG-4, and ProtecT. 

The LE for men without csPCa was estimated using data of the Dutch ERSPC [1,22]. In the period 1993–1999, a total of 21,210 men (age 54–74) were randomized to the screening arm; 19,970 men had a PSA test at the first screening round. We excluded men diagnosed with csPCa in the first round (*n* = 313) and men with life-threatening malignancies (*n* = 410), such as lung cancer, colon cancer, and leukemia. These patients should not be tested for prostate cancer, since the likelihood that they would benefit from an early PCa diagnosis is low [23]. Skin cancer was not an exclusion criterion. This led to a total of 19,247 men available for the prediction of LE for men without csPCa. Survival and follow-up time in months since time of first visit, survival status (dead or alive), age at visit, and Charlson comorbidity index were entered in a Weibull distribution model to calculate the LE for an individual without csPCa. Data for survival status was obtained by linkage with national registries (Central Bureau for Statistics, 2015). Charlson comorbidity index was missing in 158 cases and was imputed using multiple imputation with the chained equations procedure and predictive mean matching [24]. As men with a healthy lifestyle are more inclined to participate in screening studies, a healthy screenee effect may be introduced [25]. Therefore, to generalize the ERSPC data to a general Western population, the ERSPC LE was adjusted for a potential healthy screenee effect with the World Health Organization (WHO) Dutch life tables. The relative mortality between the ERSPC and the Dutch life tables was calculated with a Poisson regression corrected for age and comorbidity [26,27]. This relative mortality was added to the LE prediction for men without csPCa.

## 3. Results

For the prediction of having a biopsy-detectable csPCa, a total of 3616 men underwent sextant biopsies (PSA ≥ 3.0 ng/mL) in the first screening round ERSPC Rotterdam. A total of 313 (9%) csPCa cases were detected in addition to 572 (16%) indolent PCa cases (ISUP grade 1). Clear differences between no PCa, indolent PCa, and csPCa were noted for age, PSA, freePSA/total PSA ratio (%freePSA), prostate volume, and number of abnormal DRE/TRUS (transrectal ultrasound) findings (Table 1). Family history and IPSS did not differ between groups. The combination of PSA, age, and %freePSA (further referred to as the “basic model”) was associated with a significant increase in the concordance index compared to PSA alone (0.810 versus 0.767; *p* < 0.001), and the IPA was 21%, indicating a useful prediction model with good discrimination and calibration. The addition of DRE and a rough estimate of prostate volume to the prediction model increased the concordance index even more [to 0.839 (*p* < 0.001) and 0.862 (*p* < 0.001), respectively; Table 2]. Decision curve analysis showed a positive net benefit for all models compared to the default strategy (biopsy when PSA ≥3.0 ng/mL, Figure S1). The basic model with a 5% threshold would have a net reduction of 26% (261/1000) biopsies compared to the default strategy while not increasing the missed csPCa (Table S1). The basic model was included in the final prediction tool, as it has a good balance between high predictive accuracy and practical considerations (i.e., every GP can easily use the basic model). 

For men without csPCa, LE was estimated using a Weibull distribution on the ERSPC section Rotterdam (*n* = 19,553). The median follow-up was 15 years (interquartile range 12–17). Between 1993 and 2013, 7318 (38%) men died, 172 (2%) of whom died of PCa. The 10-year overall survival rate was 81% (95% CI: 80–81%). A healthy screenee effect with a hazard ratio of 1.6 was found between the ERSPC screening cohort versus the general population (Table S2). 

**Figure 2:**
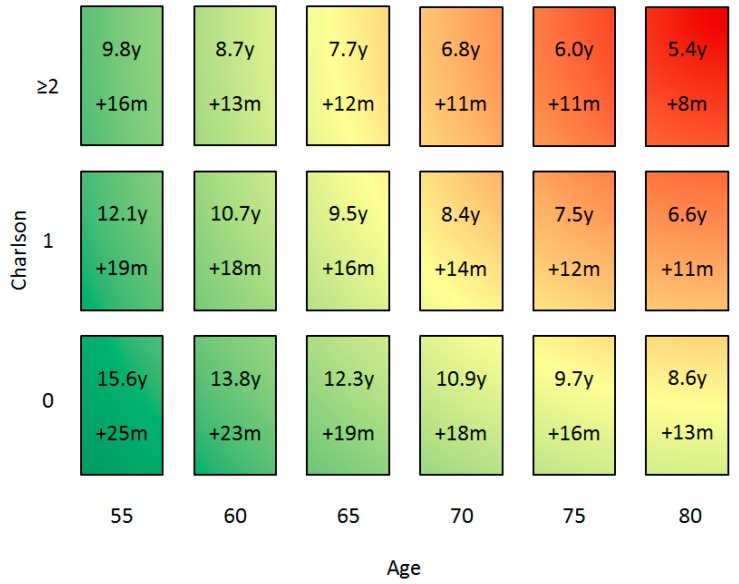
Life expectancy in years (y) for patients with clinically significant prostate cancer receiving no treatment based on Weibull distribution from the SEER. Secondly, gain in life expectancy (LE) in months (m) by csPCa treatment based on the relative risk reduction for all-cause mortality from the Prostate Cancer Intervention versus Observation Trial (PIVOT). The green color indicates a patient should be referred to a urologist, red indicates the patient should not be referred. Colors overlap because risk of csPCa on a current biopsy should also be weighted.

The estimates of LE in years for men with csPCa receiving no treatment were made on the basis of age and the Charlson comorbidity index using the SEER data (Figure 2). For men without comorbidity aged 65, 70, or 75 years, the LE was estimated to be 12.3, 10.9, and 9.7 years, respectively. 

The treatment benefit for men with csPCa was estimated as a hazard ratio (HR) of 0.84 (95% CI, 0.70 to 1.01) on all-cause mortality in the PIVOT trial [19]. This overall hazard ratio (0.84) was used to estimate the absolute treatment benefit. Treatment of csPCa was expected to increase LE with 1.6 years, 1.5 years, and 1.3 years for men without comorbidity aged 65, 70, and 75 years, respectively (Figure 2). The SPCG-4’s and ProtecT’s HR for death by any cause were 0.74 (95% CI, 0.62–0.87) and 0.93 (95% CI, 0.65–1.35), respectively. In the sensitivity analysis, shorter LE for men with csPCa receiving treatment was found when the ProtecT’s HR was used, and longer LE was found when using the SPCG-4’s HR, supplement Table S3. 

**Figure 3:**
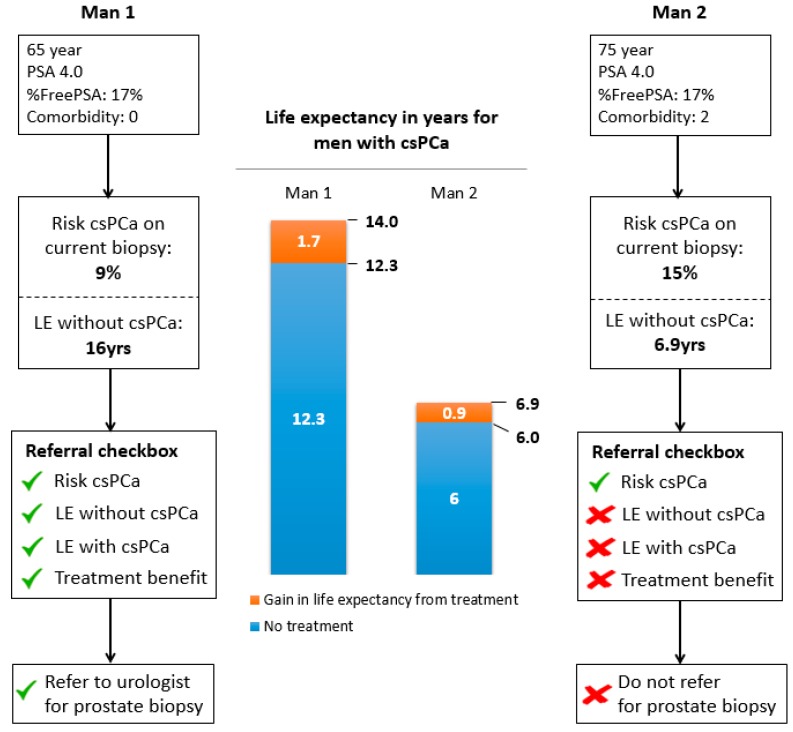
Output of the prediction tool for the general practitioner (GP). Displaying risk of clinically significant prostate cancer (csPCa; Gleason score ≥3 + 4) on a current biopsy, LE in years with and without csPCa, treatment benefit in years, and referral advice in two male examples.

The individual risk of having csPCa, the LE, and the potential absolute treatment benefit in terms of survival rate were checked against the recommendations and are summarized in an advice for referral to secondary care in Figure 3. In this example, a 65-year-old man without comorbidity had a PSA level of 4.0 ng/mL and %freePSA of 17%. His current risk of csPCa on biopsy was 9%. The patient’s life expectancy would be 12.3 years if csPCa was undetected and untreated. If the cancer was detected and treated, his life expectancy would increase by 20 months. Here, one would advise a referral for further assessment. However, a 75-year-old man with Charlson comorbidity index 2 with similar PSA and freePSA values would have a very limited absolute benefit of early detection and treatment despite a higher risk of having csPCa (15%). The latter man should not be referred to a urologist, as his potential benefit from referral would be low. 

## 4. Discussion

The integrated approach described in this manuscript provides the potential gain in LE when being diagnosed and treated for csPCa. In current practice, many men are referred with a high PSA for a prostate biopsy to the urologist, while many have benign prostatic hyperplasia. Prediction tools can already reduce unnecessary referrals for biopsies [9]. However, many old men are still referred simply on the basis of having an elevated risk of having a csPCa, while it is unlikely that they will benefit from detection and treatment of their PCa. The proposed tool can help primary care physicians triage patients for timely and necessary referral for further assessment, and as such, can aid in reducing unnecessary testing, over-diagnosis, and subsequent overtreatment. This approach can thus aid in improving the unfavorable harm–benefit ratio of opportunistic PSA testing [28]. The prediction tool is easy to use, as it requires only readily accessible information and provides risk percentages supported by recommendations on how to pursue. It is suitable for Western daily clinical practice, as it has been developed on well-known, long-term, high quality cohorts, including the SEER, the PIVOT, and the ERSPC.

Prostate cancer risk calculators including patients’ LEs have been published before [10,29]. However, the calculators lack recommendations and do not include treatment benefit in terms of overall survival. To estimate treatment benefit in the current prediction tool, PIVOT follow-up data were used [19]. The PIVOT data show that after nearly 20 years of follow-up surgery, localized PCa was associated with a lower all-cause or PCa-specific mortality compared to observation. Even with this long follow-up, the event rate was so low that no statistical significance was reached for the treatment effect of 0.84. The confidence interval indicated substantial uncertainty around this effect estimate (0.70 to 1.01). Other randomized clinical trials comparing PCa treatment with observation include the SPCG-4 and the ProtecT trial. The SPCG-4 with 29-year follow-up showed that surgery was associated with longer LE for men with localized PCa [20]. The ProtecT with 10-year follow-up found no clear differences between surgery, radiation, or active monitoring [21]. The mortality differences across these three studies may reflect differences in patients’ characteristics, the natural history of PCa, and the difference in detection and treatment methods. Sensitivity analysis was performed using the different relative treatment effects from these studies. More treatment benefit, and thus longer LE, was predicted when the relative treatment effect from the SPCG-4 was used, and shorter LE was predicted when the relative treatment of the ProtecT was used. Unfortunately, individual treatment benefit based on patients’ characteristics could not be estimated, as the numbers in all these studies are relatively small, prohibiting meaningful subgroup analysis [30]. An individual participant data meta-analysis involving the collection of the original data from the PIVOT, SPCG-4, and ProtecT would improve quality and reliability of the treatment effect estimation. This would require collaboration between researchers and take more time and resources than extracting the results from the published reports.

The construction of our model is not without its potential weaknesses. Treatment benefit is based on 20-year-old information. Our multidimensional prediction tool needs further validation based on new screening and treatment trail data. It is important to validate the contemporary treatment effect. Improvements in prostate cancer treatment might positively affect LE. Also, the predictions are limited to the information that was available at the time of analysis. For example, we did not include other predictive factors known to affect LE, e.g., marital status, body mass index (BMI), race, and smoking. The recommendation to refer a man for further assessment is based on consensus, however, this recommendation should be seen as an aid in the shared decision making process and not as a replacement. Treatment effect is based on overall treatment effect from the PIVOT, as no statistical differences in treatment effect between age groups or comorbidity categories were found. However, this may have been due to insufficient numbers to properly perform subgroup analysis. Although our prediction model only estimates LE, other outcome measures can influence a decision to refer for biopsy, e.g., quality of life, disease-free LE, or progression to metastatic disease. These other outcomes were not available in the datasets but should be considered when referring a patient. The SEER and the ERSPC data represent different settings (United States versus Europe). This might be a limitation, as the SEER consists of 15% African American men, while the ERSPC data mainly consist of Caucasian men. However, the SEER and the ERSPC have minimal selection bias and represent the general Western daily clinical practice. The field of prostate cancer detection is developing with imaging techniques such as mpMRI (multi-parametric magnetic resonance imaging) and PET-scans (positron emission tomography). MpMRI is known to detect more csPCa than TRUS-guided biopsies [31]. Therefore, the decision path might be improved with the inclusion of mpMRI target biopsies. However, mpMRI studies include referred men with a high suspicion of csPCa, which is represented by the high PSA and the high csPCa prevalence rate. Therefore, it is not yet well-established which patients should undergo an MRI, as the definition of “high risk of having csPCa” for initial men is not properly defined. Without a proper mpMRI screening trial with a standardized protocol, it is unobtainable to incorporate the mpMRI workflow in our model. In the future, our model should be validated for the prediction of csPCa with the inclusion of mpMRI and other novel biomarkers.

Our proposed GP prostate cancer prediction tool uses age, PSA, %freePSA, and comorbidity to provide recommendations to refer for prostate biopsy. These predictors provide a balance between predictive accuracy and practical considerations. Higher clinical impact can be achieved using a more accurate prediction on the risk of having csPCa when including DRE and prostate volume.

## 5. Conclusions

The estimation of life expectancy, risk of aggressive PCa, and potential benefit of prostate cancer treatment are the key aspects in the dilemma for the general practitioner and their patients regarding whether or not they should be referred for prostate biopsy. The proposed multivariable and multidimensional prediction tool needs further validation. It can provide valuable insight into the expected benefit of an early diagnosis of prostate cancer.

## Figures and Tables

**Table 1:** Characteristics of 3616 men with a biopsy stratified to prostate cancer outcome from the European Randomized Study of Screening for Prostate Cancer (ERSPC).

Characteristics	No PCa *n* = 2731 (75%)	Indolent PCa (GS 3 + 3) *n* = 572 (16%)	PCa GS ≥3 + 4 *n* = 313 (9%)
Age, years, median (IQR)	66 (60–70)	67 (61–70)	68 (64–71)
PSA, ng/mL, median (IQR)	4.0 (2.5–5.7)	5.1 (3.7–7.4)	7.8 (4.8–16.0)
%FreePSA	0.22 (0.17–0.28)	0.17 (0.12–0.24)	0.12 (0.08–0.17)
Prostate volume, mL, median (IQR)	43 (33–57)	37 (29–50)	37 (29–47)
Abnormal DRE, *n* (%)	836 (31)	236 (41)	207 (66)
Abnormal TRUS, *n* (%)	795 (29)	226 (40)	208 (66)
Positive family history, *n* (%)	210 (8)	64 (11)	30 (10)
IPSS, median (IQR)	5 (2–11)	4 (1–9)	4 (1–10)

PCa = prostate cancer, GS = Gleason Score, DRE = digital rectal exam, TRUS = transrectal ultrasound, PSA = prostate-specific antigen, IQR = interquartile range, IPSS = International Prostate Symptom Score.

**Table 2:** Concordance index corrected for optimism and index of prediction accuracy (IPA) for individual and combined predictive performance for each variable of the risk calculator predicting prostate cancer with Gleason ≥3 + 4 in 3616 men from the ERSPC.

Univariable	Concordance Index (95% CI)	IPA (%)	Multivariable	Concordance Index (95% CI)	IPA (%)
PSA	0.77 (0.74–0.80)	15.4	PSA + Age	0.77 (0.74–0.80)	15.5
Age	0.59 (0.56–0.62)	0.7	PSA + %FreePSA	0.80 (0.77–0.83)	20.3
%FreePSA	0.78 (0.75–0.81)	11.1	PSA + Age + %FreePSA	0.81 (0.78–0.84)	21.0
DRE	0.67 (0.64–0.70)	3.9	PSA + Age + DRE	0.82 (0.79–0.84)	22.3
Prostate volume	0.60 (0.56–0.63)	0.7	PSA + Age + DRE + %FreePSA	0.84 (0.81–0.86)	26.3
FH	0.51 (0.49–0.52)	0.0	PSA + Age + DRE + %FreePSA + PV	0.86 (0.84–0.88)	28.3
IPSS/AUA	0.52 (0.49–0.56)	0.0	Above + TRUS	0.87 (0.85–0.90)	31.6
TRUS	0.68 (0.65–0.71)	4.5	Above + FH and IPSS/AUA	0.86 (0.84–0.88)	31.6

%FreePSA = FreePSA divided by PSA, AUA = American Urological Association symptom score, PV = prostate volume, FH = family history.

## Supplementary Materials

The following are available online at https://www.mdpi.com/2075-4426/9/2/19/s1, Figure S1: Decision curve analysis for different prediction models incorporating prostate measurements, Table S1: Clinical impact of the three prostate cancer risk calculators, Table S2: Characteristics of 19,247 men from the screening arm of the ERSPC section Rotterdam stratified to alive and death during the period 1993–2013, Table S3: Characteristics of the ERSCP screenings arm vs. the Dutch general population.
