# Supplementary material for: A Tool for Shared Decision Making on Referral for Prostate Biopsy in the Primary Care Setting: Integrating Risks of Cancer with Life Expectancy
Source: J Pers Med. 2019 Apr 22;9(2):19. doi: 10.3390/jpm9020019 (PMC6617187; doi:10.3390/jpm9020019)
Supplement: Supplementary file 1 [file jpm-09-00019-s001.pdf]

Supplements

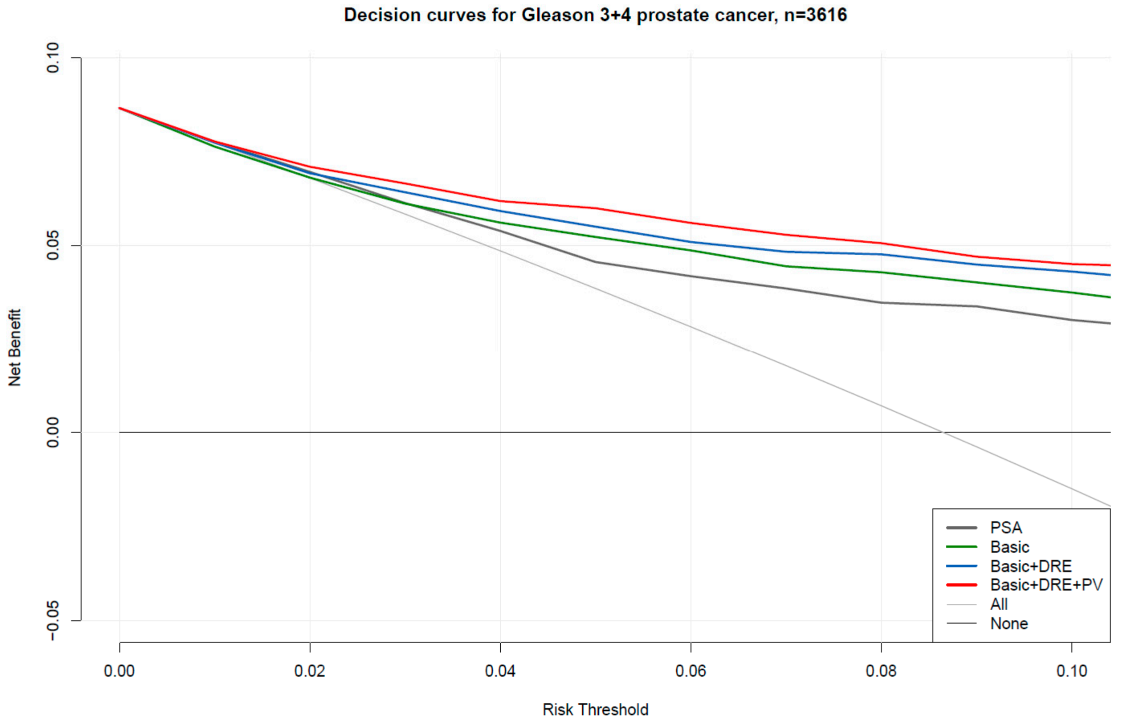

Figure S1. Decision curve analysis for different prediction models incorporating prostate measurements in 3616 patients from ERSPC.

Table S1. Clinical impact of the three prostate cancer risk calculators.

Net benefit of the three prostate cancer prediction models for the general practitioner																
**Threshold**	**Basic model: PSA+FreePSA+Age**					**Basic + DRE**					**Basic+DRE+PV(DRE)**					**Ref. strategy**
	A	B	C	ΔD	E	A	B	C	ΔD	E	A	B	C	ΔD	E	D
Total, n		87 (0)	158 (0)				87 (0)	158 (0)				87 (0)	158 (0)			
3%	320	7 (8)	27 (17)	3	90	428	7 (8)	41 (26)	6	188	466	6 (7)	48 (31)	8	263	58
**5%**	**526**	**13 (15)**	**57 (36)**	**14**	**261**	**606**	**15 (17)**	**70 (44)**	**17**	**313**	**622**	**11 (13)**	**67 (43)**	**22**	**406**	**38**
10%	770	30 (34)	101 (64)	52	471	787	27 (31)	102 (65)	58	521	791	25 (29)	97 (61)	60	539	-15

A: Absolute number of biopsy reduction per 1000

B: Absolute number of missed PCa GS ≥3+4 per 1000 (% of total PCa GS ≥3+4)

C: Absolute number of missed indolent PCa per 1000 (% missed of total indolent PCa)

D: Net benefit x 1000; ΔD: Additional net true positives per 1000 men compared with reference strategy

E: Net reduction of biopsies per 1000 men, compared to the reference strategy (biopsy is advised when PSA >3.0ng/ml); formula: ΔNB/(threshold/(1-threshold))*1000.

Table S2. Characteristics of the ERSCP screenings arm vs. 1458 men from the Dutch general population (year 2015).

	**ERSPC**<br>**n=19247**	**General Dutch Population**<br>**n=1458**
**Age, median (IQR)**	63 (59-68)	66 (60-72)
**Charlson comorbidity index**		
**0**	14095 (73)	801 (55)
**1**	4332 (23)	457 (31)
**2**	662 (3)	200 (14)
**missing**	158 (1)	-

Table S3. Sensitivity analysis of the life expectancy of men with clinical significant prostate cancer receiving prostate cancer treatment using the different treatment hazard ratios in the prostate cancer treatment trials.

Life expectancy in years
Charlson comorbidity score = 0

Age	Treatment - Hazard rate (95% CI)			
	None	ProtecT	PIVOT	SPCG-4
		0.93 (0.65 – 1.35)	0.84 (0.70 - 1.01)	0.74 (0.62 – 0.87)
**55**	15.6	16.3	17.7	19.4
**65**	12.3	12.8	13.9	15.3
**75**	9.7	10.1	11.0	12.0

Life expectancy in years
Charlson comorbidity score = 1

Age	Treatment - Hazard rate (95% CI)			
	None	ProtecT	PIVOT	SPCG-4
		0.93 (0.65 – 1.35)	0.84 (0.70 - 1.01)	0.74 (0.62 – 0.87)
**55**	12.1	12.6	13.7	15.0
**65**	9.5	9.9	10.8	11.8
**75**	7.5	7.8	8.5	9.3

Life expectancy in years
Charlson comorbidity score ≥ 2

Age	Treatment - Hazard rate (95% CI)			
	None	ProtecT	PIVOT	SPCG-4
		0.93 (0.65 – 1.35)	0.84 (0.70 - 1.01)	0.74 (0.62 – 0.87)
**55**	9.8	10.2	11.1	12.1
**65**	7.7	8.0	8.7	9.5
**75**	6.0	6.3	6.9	7.5
